# The impact of COVID-19 on the WHO FCTC, cessation, and tobacco policy

**DOI:** 10.18332/tid/130779

**Published:** 2020-12-08

**Authors:** Laurent Huber, Ubaldo Cuadrado, Raquel Fernandez-Megina, Martin Raw, Adriana Blanco Marquizo, Kelsey Romeo-Stuppy

**Affiliations:** 1 Action on Smoking and Health, Washington, United States; 2 Nofumadores.org, Madrid, Spain; 3 School of Global Public Health, New York University, New York, United States; 4 WHO Framework Convention on Tobacco Control, Geneva, Switzerland

**Keywords:** COVID-19, WHO FCTC, cessation, tobacco policy

## Abstract

‘In the absence of the COVID-19 pandemic, many people in tobacco control worldwide would have been at the Hague, Netherlands, from 9–14 November for the 9th Conference of the Parties (COP9) of the WHO Framework Convention on Tobacco Control (WHO FCTC), advocating for even stronger policies against the tobacco epidemic. The COP has been postponed to 2021, but the pandemic did not stop the global civil society from “virtually” gathering to talk about the WHO FCTC, where it is and where it is going.’

Even in ‘normal’ times, the tobacco industry’s marketing and selling of addictive, lethal products like cigarettes is a leading cause of global illness, resulting in more than 8 million deaths per year. Consequently, they are a major drain on national health systems^1^. This negative impact on society is further exacerbated by the COVID-19 pandemic.

After conducting an extensive review of the literature, the World Health Organization released a scientific brief on 30 June in which they concluded that ‘at the time of this review, the available evidence suggests that smoking is associated with increased severity of disease and death in hospitalized COVID-19 patients’^2^. This puts smokers at increased risk during the pandemic. Furthermore, given the growing scientific evidence that COVID-19 is transmitted through aerosols^3^, we should keep tobacco in mind when taking measures to stop the COVID-19 pandemic.

Thus, it is critical that countries intensify and accelerate implementation of the WHO Framework Convention on Tobacco Control^4^. For example, robust implementation of price and tax measures, mandated in Article 6 of the WHO FCTC, will not only decrease smoking prevalence^5^ but also will generate much needed funding during the economic crisis caused by COVID-19. To date, according to the WHO Report on the Global Tobacco Epidemic, 2019, only 38 countries have achieved the benchmark of taxes representing 75% or more of final retail price^6^.

Furthermore, while many countries have reported that an increasing number of smokers have expressed an interest in quitting smoking, access to cessation support is highly uneven around the world. According to a 2017 survey on progress in implementation of Article 14 of the WHO FCTC, on tobacco dependence and cessation, two-thirds of the 142 countries that participated in the survey did not even have an official national treatment strategy^7^. As a result, many smokers are unable to get the support they need to quit.

Some countries, like Spain for example, have taken some positive steps to protect their citizen’s health by extending smoke-free policies to outdoor areas during COVID-19^8^. But the Spanish government, as well as all governments around the world, could and should do more to take advantage of the WHO FCTC as a tool to advance globally agreed health, development and human rights objectives. This is particularly critical during and after the COVID-19 pandemic.
